# Parathyroid adenomas – a cluster of boys

**DOI:** 10.1186/1687-9856-2015-S1-P60

**Published:** 2015-04-28

**Authors:** Angela Titmuss, Shubha Srinivasan, Paul Benitez-Aguirre, Albert Shun, Ann Maguire, Craig Munns, Chris Cowell, Geoff Ambler, Kim Donaghue

**Affiliations:** 1 Institute of Endocrinology and Diabetes, The Children’s Hospital Westmead, Sydney, NSW, Australia; 2 Discipline of Paediatrics and Child Health, University of Sydney, Sydney, NSW, Australia; 3 Department of Surgery, The Children’s Hospital Westmead, Sydney, NSW, Australia

## 

Primary hyperparathyroidism is rare in children and adolescents, representing 1% of all cases, with a slight female preponderance [1-3]. 3-5% of cases are hereditary and may represent the initial clinical manifestation of multiple endocrine neoplasia type 1 (MEN1).

Over the last 30 years, ten cases of primary hyperparathyroidism have presented to our hospital, all aged 11-14 years, with nine cases being male, and eight cases over the last six years. Presenting features included headache and blurred vision in five patients, abdominal pain and nausea (in three), renal calculi (in four), generalized bone pain (in three) and two asymptomatic. One patient had received radiation for acute lymphoblastic leukaemia. Other history included ADHD (in one patient), mild developmental delay (in one), depression (in one) and fine motor difficulties (in one). No patients had a significant family history.

Peak corrected calcium level ranged between 3.12-3.65 mmol/L (2.1-2.65), peak PTH level 6.9-154 pmol/L (1-7), and urine calcium creatinine ratio 0.47-2.76mM/mM (0.04-0.7). Serum alkaline phosphatase was 212-549U/L (80-355), and normal in three of the renal calculi patients. Bony changes were seen in three patients, with flaring of clavicles and widening of epiphyses, decreased phalangeal cortical density and osteopenia. 25-OH calciferol was low at 27-39nmol/L (>50) in 3 of 7 patients in whom it was measured.

Thyroid ultrasound detected suspicious lesions in seven patients and was normal in three patients. Sestamibi scan was negative for three patients (with one ectopic gland) and indicated a single overactive gland in seven patients. All patients had a positive result from at least one modality.

All patients underwent surgical resection, with a single benign parathyroid adenoma identified in each case. The only significant post-operative issues were initial hypocalcaemia (lowest cCa 1.8mmol/L) in eight patients, requiring management with calcitriol and elemental calcium. There was normalisation of calcium over several weeks post-operatively, with gradual weaning of supplementation required. In all, post-operative PTH level was suppressed. MEN1 screening has been negative for all patients.

This case series illustrates the difficulties involved in the diagnosis of parathyroid adenoma in children, requiring both scintigraphy and ultrasound. Scintigraphy has been reported in the literature to have 88% sensitivity, lower in our case series, compared to 78% sensitivity for ultrasound [4]. The gender mix of our cases differs significantly from other reported case series, being predominantly male [3,5] and there is a suggestion of increasing incidence over recent years.
